# Stem cells and bio scaffolds for the treatment of cardiovascular diseases: new insights

**DOI:** 10.3389/fcell.2024.1472103

**Published:** 2024-12-12

**Authors:** Zahra Sadat Razavi, Simin Farokhi, Golnaz Mahmoudvand, Arian Karimi-Rouzbahani, Bahareh Farasati-Far, Samaneh Tahmasebi-Ghorabi, Hamidreza Pazoki-Toroudi, Masoud Saadat-Fakhr, Hamed Afkhami

**Affiliations:** ^1^ Physiology Research Center, Iran University of Medical Sciences, Tehran, Iran; ^2^ Student Research Committee, USERN Office, Lorestan University of Medical Sciences, Khorramabad, Iran; ^3^ Department of Chemistry, Iran University of Science and Technology, Tehran, Iran; ^4^ Master of Health Education, Research Expert, Clinical Research Development Unit, Emam Khomeini Hospital, Ilam University of Medical Sciences, Ilam, Iran; ^5^ Faculty of Medicine, Tehran Medical Sciences Branch, Islamic Azad University, Tehran, Iran; ^6^ Cellular and Molecular Research Center, Qom University of Medical Sciences, Qom, Iran; ^7^ Nervous System Stem Cells Research Center, Semnan University of Medical Sciences, Semnan, Iran; ^8^ Department of Medical Microbiology, Faculty of Medicine, Shahed University, Tehran, Iran

**Keywords:** cardiovascular heart disease, heart failure, stem cell, cell therapy, bio scaffold

## Abstract

Mortality and morbidity from cardiovascular diseases are common worldwide. In order to improve survival and quality of life for this patient population, extensive efforts are being made to establish effective therapeutic modalities. New treatment options are needed, it seems. In addition to treating cardiovascular diseases, cell therapy is one of the most promising medical platforms. One of the most effective therapeutic approaches in this area is stem cell therapy. In stem cell biology, multipotent stem cells and pluripotent stem cells are divided into two types. There is evidence that stem cell therapy could be used as a therapeutic approach for cardiovascular diseases based on multiple lines of evidence. The effectiveness of stem cell therapies in humans has been studied in several clinical trials. In spite of the challenges associated with stem cell therapy, it appears that resolving them may lead to stem cells being used in cardiovascular disease patients. This may be an effective therapeutic approach. By mounting these stem cells on biological scaffolds, their effect can be enhanced.

## Introduction

Despite great progress in medical research over the past few decades, cardiovascular diseases (CVDs) remain the primary cause of mortality and morbidity globally, imposing a substantial burden on global health. Approximately 30% of worldwide mortality is attributed to cardiovascular diseases (CVDs), rendering them one of the most urgent public health concerns of our era. Projections from the World Health Organization (WHO) and the American Heart Association indicate that by 2030, the number of deaths caused by cardiovascular diseases would exceed 23 million per year. The presented data highlights the increasing severity of the problem and emphasizes the pressing requirement for creative approaches to decrease the occurrence and consequences of cardiovascular disease (Di Cesare et al., 2024; Correction to: heart disease and stroke statistics—2023 update: a report from the American heart association, 2023; Xu et al., 2022).

In addition to becoming a health concern, the increasing prevalence of cardiovascular illnesses imposes a significant economic cost. The expenses related to the treatment of cardiovascular diseases (CVDs) are immense, amounting to billions of dollars each year on healthcare services, hospital stays, drugs, and long-term care. As populations age and the prevalence of risk factors such as obesity, diabetes, and hypertension increases, the economic burden is anticipated to deteriorate. Furthermore, the financial strain is exacerbated by the indirect expenses linked to decreased production and the extended care needed for patients who survive myocardial infarctions, cerebrovascular accidents, or suffer from chronic heart failure (Brown et al., 2020). Based on these considerations, the advancement of treatment alternatives that are more economical, effective, and less intrusive has become a crucial focus for healthcare systems globally (Csöbönyeiová et al., 2022; Ge et al., 2023). Heart failure, a medical disorder characterized by a significant impairment in the heart’s capacity to efficiently circulate blood, is a primary factor contributing to hospitalization and mortality in individuals with cardiovascular disease (CVD). Although pharmaceutical medicines have made significant progress and mechanical support devices like ventricular assist devices (VADs) have been developed, heart transplantation is still the only definitive therapy for people suffering from end-stage heart failure (Fadini et al., 2020). Yet, heart transplantation is riddled with obstacles, such as a critical scarcity of donor organs, the requirement for lifelong immunosuppression, and the possibility of transplant rejection. The aforementioned constraints emphasize the necessity for alternative therapeutic approaches that can effectively target the root causes of heart failure and provide enduring advantages without the consequent hazards of transplantation (Haba et al., 2021; Feng et al., 2024).

Recent years have seen the emergence of stem cell therapy as a promising method for treating cardiac illness, including heart failure. Stem cells inherently possess the distinctive capacity to undergo differentiation into many cell types, including cardiomyocytes, the cells accountable for the contractile activity of the heart. Two kinds of stem cells now under investigation for cardiac treatment, resident cardiac stem cells (CSCs) and induced pluripotent stem cells (iPSCs), have demonstrated significant promise. cardiac stem cells (CSCs), located in the heart, has the capacity to undergo differentiation into several types of cardiogenic cells, therefore presenting a promising opportunity for the regeneration of impaired cardiac tissue. Likewise, induced pluripotent stem cells (iPSCs), produced by reprogramming mature cells to exhibit pluripotency, may be manipulated to differentiate into cardiac cells, offering a flexible and individualized therapeutic alternative (Golpanian et al., 2016; Hu and Feng, 2017; Huang et al., 2024; Farokhi et al., 2024). Numerous clinical trials have investigated different sources of stem cells, such as skeletal myoblasts, bone marrow mononuclear cells (BMMNCs), and more recently, induced pluripotent stem cells (iPSCs), to assess their capacity to restore and regenerate cardiac tissue. Experimental studies have demonstrated that stem cells have the potential to boost cardiac function, diminish scar tissue, and facilitate the general recuperation of individuals afflicted with heart illness. However, the therapeutic use of stem cells in the treatment of cardiovascular disorders is still in its nascent phase, and various obstacles persist (Xu et al., 2002). One of the primary challenges is to determine the optimal stem cell type and delivery technique to obtain reliable and enduring outcomes. Furthermore, there are apprehensions regarding the possible hazards linked to stem cell treatment, such as the development of malignancies or undesired immunological reactions, which must be duly tackled by meticulous clinical experimentation (Gallina et al., 2015; Simon et al., 2008).

**FIGURE 1:**
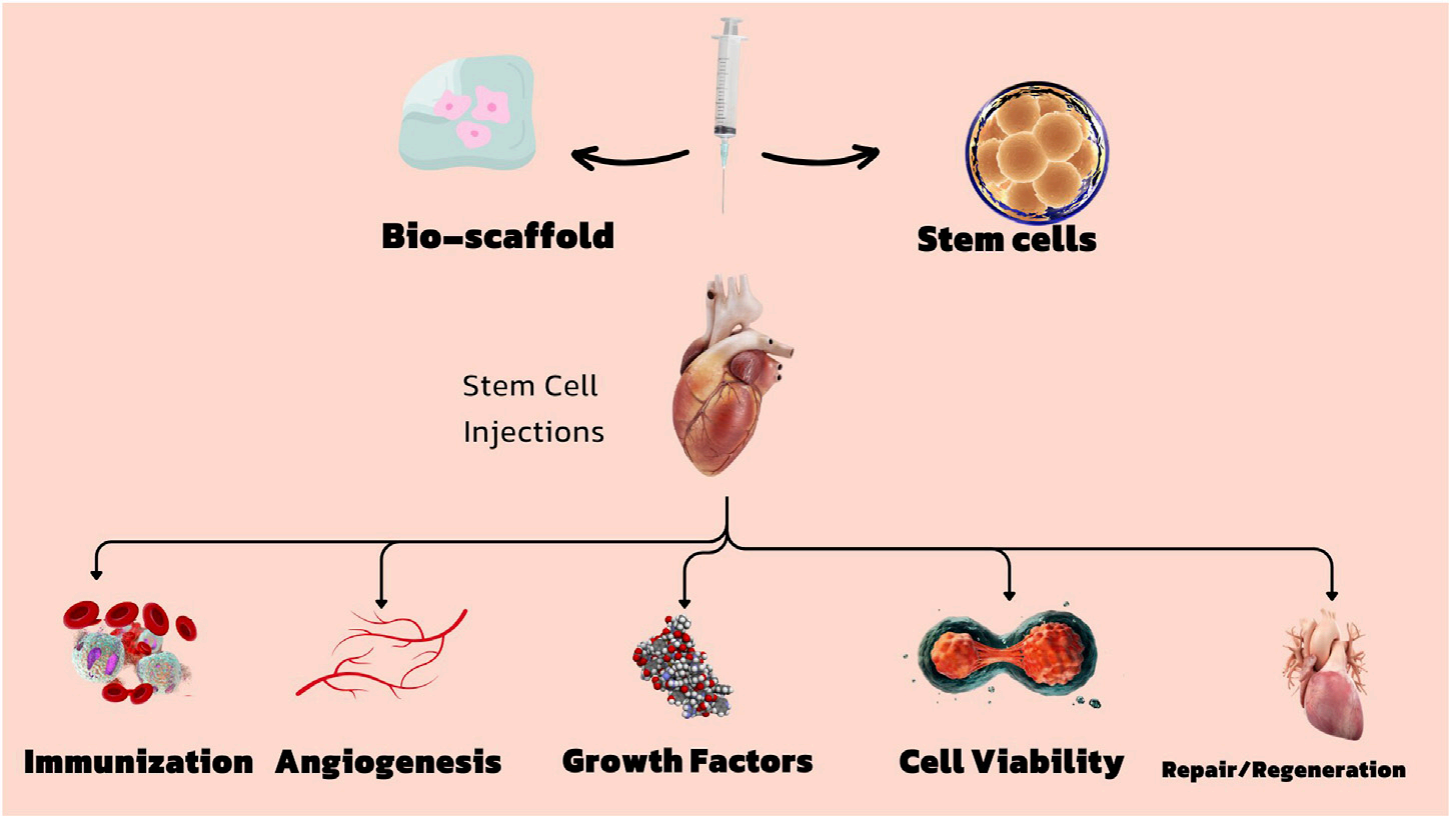
Stem cells and bio scaffolds for the treatment of cardiovascular diseases.

Furthermore, although stem cell treatment has significant potential, it is not devoid of its constraints. One example is the restricted availability of very desirable stem cell characteristics, especially in the context of adult-derived stem cells, which often decrease in quantity and effectiveness as they age. Recent studies have demonstrated that a mere 1%–2% of cardiac c-kit + cells exhibit the requisite multifunctional capability for efficient cardiac repair, rendering them scarce and difficult to separate. Moreover, research has shown that the levels of cardiac progenitor cells (CPCs) in humans decline considerably beyond the age of two, therefore restricting their potential for therapeutic applications in later stages of life (Fathi et al., 2020; Yu et al., 2022). The reduction associated with aging poses a substantial challenge in using these cells for the treatment of cardiac diseases in elderly people, who are the most likely to get benefits from such treatments (Guo et al., 2020; Trzyna and Banaś-Ząbczyk, 2021; Zhao Y. et al., 2021a). This study explores the growing significance of bio-scaffolds in combination with stem cells for the management of cardiovascular disorders, providing novel perspectives on how these technologies might be employed to improve therapeutic results. Biological scaffolds offer a supporting structure that can direct the development, specialization, and incorporation of stem cells into injured cardiac tissue, therefore enhancing the effectiveness of stem cell-based treatments. Through the emulation of the extracellular matrix of the heart, these scaffolds have the ability to establish a more advantageous milieu for tissue regeneration, therefore facilitating enhanced integration of the transplanted cells and ultimately enhancing the total structural and functional restoration of the heart. Continued progress in research in this field suggests that the integration of stem cells and bio-scaffolds has the potential to completely transform the management of heart failure and other cardiovascular disorders, offering fresh optimism for patients with few treatment alternatives (Figure 1).

### Types of cells

During reimplantation, autologous cells are obtained from the same individual (Simeon et al., 2021). The least susceptible to pathogen rejection and transmission are autologous cells, however these are occasionally unavailable. For instance, hereditary illness lacks adequate autologous cells (Ma et al., 2017). Moreover, patients with severe burns, the older, or those who are severely ill may not have sufficient autologous cells to create the cell lines (Eschenhagen and Carrier, 2019). Concerns exist over the necessity of such procedures due to the fact that certain cell types must be removed from the patient and could result in persistent discomfort or donor site infection (Palà et al., 2020). Before usage, autologous cells must also be cultivated from specimens: Autologous solutions could not be particularly quick because this could take some time (Moghaddam et al., 2019). The use of bone marrow and adipocyte mesenchymal stem cells has lately become popular. These cells can develop into many tissues, such as bone, cartilage, fat, and nerve. Fat may be swiftly and readily used to separate many cells, opening up the possibility of producing plenty of cells in a hurry (Henning, 2021; Andalib et al., 2023).

Allogeneic cells are those that originate from a single donor. It has been shown that the use of dermal fibroblasts from human skin is a risk-free and, as a result, acceptable choice for the engineering of skin tissue. This is despite the fact that there are ethical restrictions placed on the utilization of human cells in the context of laboratory research (Xia et al., 2020).

These xenogeneic cells cannot be compared to those of any other species. Particularly animal cells have just seen extensive application in research efforts directed towards the development of cardiovascular implants (Moghaddam et al., 2019).

Syngeneic cells, also known as isogenic cells, are those that have been extracted from genetically identical creatures like twins, clones, or carefully researched animal models (Csöbönyeiová et al., 2022).

There is just one creature that has primary cells. There is only 1 cell present in each of the secondary cells (Eschenhagen and Carrier, 2019). The choice of stem cell source and differentiation for therapeutic purposes is determined by the specific medical condition being targeted. Multiple types of stem cells, such as embryonic, adult, and induced pluripotent stem cells, have been extensively studied for their potential in therapy. Every type has its own set of benefits and constraints (Thanaskody et al., 2022).

#### Best source for stem cells

Embryonic Stem Cells (ESCs): These stem cells are derived from embryos and have the ability to develop into different cell types. These cells possess the remarkable ability to transform into various cell types within the body, which greatly enhances their significance in the field of regenerative medicine. Nevertheless, their utilization raises ethical concerns Adult stem cells are a remarkable type of cells that exist in various parts of the body. They possess the incredible ability to divide and transform into different cell types, allowing them to replace dying cells and repair injured tissues (Vazin and Freed, 2010). They are widely accepted and have been utilized in a range of therapies, including bone marrow transplants. Induced Pluripotent Stem Cells (iPSCs) are adult cells that have undergone reprogramming to acquire a state similar to embryonic stem cells. They have the potential to be tailored to individual patients, which can help minimize the chances of immune rejection. However, additional research is needed to ensure the safety and effectiveness of their clinical use (Chang et al., 2019).

#### Maximizing differentiation

The differentiation of stem cells into specific cell types is essential for their therapeutic efficacy. As an illustration, in the treatment of neurodegenerative diseases, it is desirable to differentiate stem cells into neurons or glial cells (Mustafa et al., 2023). On the other hand, in the context of heart disease, the focus is on differentiating them into cardiac muscle cells. When selecting a stem cell source and differentiation method, it is crucial to consider their demonstrated effectiveness for the specific condition being targeted (Augustine et al., 2021).

Ensuring safety is of utmost importance when evaluating the stem cell type and the differentiation process, as it helps to minimize any potential side effects.

**FIGURE 2:**
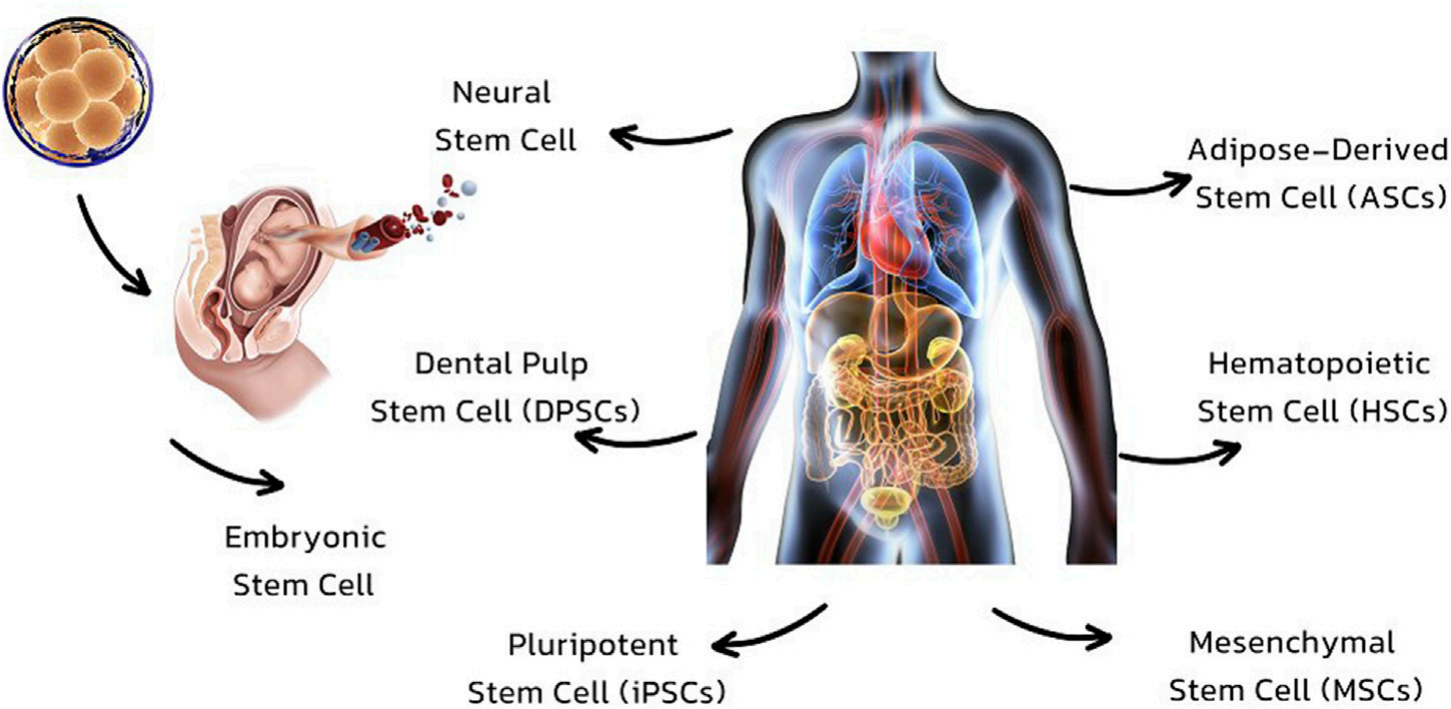
Types of stem cells which are used in regeneration and repair of organs.

Consideration should be given to the ethical and legal regulations regarding the use of various stem cell types (Fadini et al., 2020; Rigato and Fadini, 2018; Mahjoor et al., 2021) (Figure 2). In the table below, the types of stem cells and their different characteristics are listed in full (Table 1).

**TABLE 1:** Types of stem cells, sources, advantages and disadvantages.

Type of stem cell	Source	Advantage	Disadvantage	Potential applications	Ethical concerns	Challenges in therapy	References
Embryonic Stem Cell	Embryo (embryoblast)	Pluripotent, capable of differentiating into any cell type	Ethical problems, safety concerns, tissue availability	Regenerative medicine, disease modeling, drug discovery	High ethical concerns	Tumorigenesis, immune rejection	Zakrzewski et al. (2019)
Fetal Neural Stem Cell	Fetus	Multipotent (for neural cell types), useful for neural regeneration	Ethical problems, safety concerns, tissue availability, difficulty in directing relevant differentiation	Neural tissue engineering, treatment of neurodegenerative diseases	High ethical concerns	Directing differentiation	Bruno et al. (2024)
Adult Neural Stem Cell	Adult tissue (CNS)	Multipotent (for neural cell types), ethically favorable, autologous-based therapy	Safety concerns, difficult to extract	Repair of CNS injuries, neurodegenerative disease treatment	Low ethical concerns	Extraction, limited plasticity	Cecerska-Heryć et al. (2023)
Adult Non-Neural Stem Cell	Adult tissue (various)	Ethically favorable, autologous-based therapy, tissue availability	Safety concerns, lineage restricted	Regeneration of specific tissues like bone, cartilage, or muscle	Low ethical concerns	Limited differentiation potential	Atia et al. (2024)
Induced pluripotent stem cells (iPSCs)	Adult tissue (various)	Ethically favorable, autologous-based therapy, tissue availability, pluripotent	Safety concerns	Regenerative medicine, personalized medicine, drug screening	Minimal ethical concerns	Tumorigenesis, instability in culture	Madrid et al. (2024)
Mesenchymal Stem Cell (MSCs)	Bone marrow, adipose tissue, umbilical cord	Ethically favorable, immunomodulatory properties, supports tissue repair	Limited differentiation potential, risk of unwanted immune reactions	Bone and cartilage repair, treatment of inflammatory diseases	Low ethical concerns	Limited lifespan, senescence	Pittenger et al. (2019)
Hematopoietic Stem Cell (HSCs)	Bone marrow, peripheral blood, umbilical cord blood	Replenishes blood cells, widely used in transplantation	Limited differentiation (blood cells only), risk of graft-vs-host disease	Treatment of blood disorders like leukemia, lymphoma	Low ethical concerns	Graft-vs-host disease	(Bello et al., 2018)
Amniotic Fluid Stem Cell	Amniotic fluid	Ethically favorable, high proliferation rate, multipotent	Limited availability, ethical concerns related to extraction	Prenatal diagnosis, regenerative medicine	Moderate ethical concerns	Limited availability	Rosner et al. (2023)
Cancer Stem Cell	Tumor tissues	Potential target for cancer therapy, resilient cell population	High resistance to conventional therapies, ethical concerns in research	Cancer treatment, drug resistance studies	High ethical concerns	Resistance to conventional therapies	Alnasser (2023)
Adipose-Derived Stem Cell (ASCs)	Adipose tissue	Abundant source, less invasive extraction, multipotent	Limited differentiation capacity	Tissue engineering, regenerative medicine	Low ethical concerns	Lineage restrictions	Biniazan et al. (2024)
Dental Pulp Stem Cell (DPSCs)	Dental pulp (extracted teeth)	High proliferative ability, multipotent	Limited differentiation potential, extraction difficulties	Dental tissue regeneration, bone repair	Low ethical concerns	Extraction, limited applications	Fujii et al. (2023)

### Stem cells for tissue engineering

In selecting an appropriate biological cellular material, also known as scaffolding, one of the most critical aspects to focus on is choosing the cell type that is most ideal for tissue engineering. (SCs), also known as a source of cells capable of developing into cardiac muscle/cardiomyocytes (CMCs), smooth muscle cells (SMCs), and endothelial cells (ECs), have been shown to be able to repair heart tissue. They play an important part in the study of TE due to the qualities that have been listed (Wang et al., 2021).

#### Embryonic stem cell

Embryonic stem cells, often known as ESCs, are one of the cellular sources that are applied in techniques involving TE. Internal cell mass of the preimplantation blastocyst is the source of embryonic stem cells (ESCs) (Nasser et al., 2020). They are capable of differentiating into a variety of three-layer germ cells of varying kinds. The use of human embryonic stem cells (hESC) may be advantageous in the field of tissue engineering for the heart. HESC-derived cardiomyocytes (hESC-CMC) demonstrated extremely good characteristics in a research carried out by Landry et al. These phenotypes included myofibril alignment, density, morphology, contractile performance, and gene expression profiles. Nevertheless, only laboratory cultures were validated after days *in vitro* (Lundy et al., 2013). According to the findings given in a research by Henning (2021), several experiments have been carried out to validate the effective differentiation of embryonic stem cells (ESCs) into cardiomyocytes. The researchers Andalib et al. (2023) investigated how the native cardiac ECM might influence the differentiation of hESC. After the extraction of cardiac ECM from the digested pig hearts, the cardiac ECM was combined with collagen and used to generate hydrogels for use in cell culture. When compared with HESCs grown on less ECM hydrogels, HESCs cultured on biomass material consisting of 75% ECC of native goats and 25% hydrogels with added growth factors did not demonstrate adequate differentiation with cardiac T and troponin expression and contractile function. Native. ESCs have been shown to be a viable treatment option for cardiac TE, according to a number of studies (Xiao et al., 2021). In addition, some factors such as ethical concerns, immune stimulation and the risk of tumorigenesis make ESCs a very inconsistent choice of cell source for TE (Nugraha et al., 2019) (Table 2).

**TABLE 2:** Application and aspects of Embryonic stem cell.

Aspect	Details	Advantages	Disadvantages	Research insights	Cardiomyocyte functionality	Long-term viability	Differentiation efficiency	Scalability	Integration with host tissue	Tumor suppression strategies	Ethical debate resolution
Source of ESCs	Derived from the inner cell mass of the preimplantation blastocyst	High pluripotency, allowing differentiation into various cell types, essential for regenerative applications like cardiac tissue engineering	Ethical concerns due to the destruction of embryos; regulatory challenges and public opposition in some regions	ESCs are highly efficient in producing cardiomyocytes, essential for heart repair, but their use is heavily regulated due to ethical concerns	ESC-derived cardiomyocytes can replicate the electrophysiological and contractile properties of native heart cells	Promising results in maintaining the viability and function of transplanted tissues over extended periods	High, with ongoing improvements in protocols to increase the efficiency of differentiation into specific cell types like cardiomyocytes	ESCs can be expanded in large quantities, but scaling up for clinical applications remains challenging	ESC-derived tissues show potential for integration with native heart tissues, but further research is needed to optimize this process	Advances in gene editing and cell sorting are being used to minimize the risk of tumor formation from undifferentiated cells	Development of alternative stem cell sources like iPSCs is helping to reduce the ethical issues associated with ESC use
Differentiation Potential	ESCs can differentiate into multiple germ layers, giving rise to a variety of cell types, including cardiomyocytes	Versatile for generating different cardiac cell types, crucial for heart tissue repair and engineering	Risk of uncontrolled differentiation, leading to the formation of teratomas (tumors), which complicates clinical applications	Methods to control ESC differentiation, such as electrical stimulation or chemical induction, are under investigation to increase the efficiency and safety of generating specific cardiac cells	ESC-derived cardiomyocytes can exhibit properties like proper beating patterns and ion channel expression, essential for functional heart tissue	Studies focus on the survival of ESC-derived tissues *in vivo*, with encouraging outcomes in terms of longevity and integration	Protocols are being optimized to increase the yield of functional cardiomyocytes from ESCs	While scalable, maintaining consistency and functionality at large scales is a key challenge	Effective integration is crucial for restoring heart function, with research focusing on improving the mechanical and electrical coupling of transplanted tissues	Strategies include pre-differentiation of ESCs into committed lineages before transplantation to reduce the risk of tumorigenesis	Public debates and ethical guidelines are shaping the responsible use of ESCs, with iPSCs providing a promising alternative
Cardiac Tissue Engineering Applications	ESCs have been used to generate engineered heart tissues (EHTs) that mimic the structure and function of native cardiac tissue	Ability to regenerate damaged myocardium, potentially restoring heart function after injury	Limited *in vivo* validation; challenges in integrating engineered tissues with native heart tissue; potential immune rejection	Recent studies demonstrate that ESC-derived cardiac tissues show promising results in lab settings, but further research is needed to confirm their effectiveness in living organisms	ESC-derived tissues can function similarly to native heart tissue, including appropriate response to electrical stimuli	Long-term studies are crucial for assessing the durability of ESC-derived tissues post-transplantation	Enhanced differentiation protocols are being developed to ensure that ESCs consistently produce high-quality cardiac cells	Bioreactors and automated systems are being explored to improve the scalability of ESC-derived tissues for therapeutic use	Research is focused on improving the structural and functional integration of ESC-derived tissues with the host myocardium	The use of tumor suppressor genes and selective differentiation markers is being explored to enhance the safety of ESC-based therapies	Continuous refinement of ethical standards and alternative approaches, such as iPSCs, are helping to address the moral concerns related to ESC use

#### Mesenchymal stem cells

Bone marrow, adipose tissue, tonsil tissue, and mesenchymal stem cells (MSCs) are some of the tissues from which mesenchymal stem cells can be isolated (Liu et al., 2022; Mahjoor et al., 2023a; Mahjoor et al., 2023b; Fakouri et al., 2024). Under normal culture conditions, MSCs have plastic adherent properties and show characteristics similar to fibroblasts in terms of their morphology. As well as CD73, CD90, and CD105, there are specific cell surface markers expressed on them. In addition to self-renewing cells, MSCs also can differentiate into a wide range of mesodermal lineages, including adipocytes, muscles, chondrocytes, and osteoblasts (Mazini et al., 2020; Mirshekar et al., 2023). Furthermore, there is growing evidence that MSCs possess both immune-modulatory functions and pro-antigenic activity, which are beneficial for tissue regeneration, in addition to their differentiation potential (Jiang and Xu, 2020; Mahmoudvand et al., 2023). Through the secretion of various immune-modulatory cytokines, MSCs impair dendritic cell and T-cell function in addition to generating an immunosuppressive environment at the local level (Fu et al., 2019; Mahjoor et al., 2023b). As a matter of fact, MSCs have been shown to promote angiogenesis by secreting factors that promote angiogenesis (Andrzejewska et al., 2019). The use of MSCs in treating a wide range of human diseases has been investigated through clinical trials worldwide. These include cardiovascular diseases, bone and cartilage diseases, neurological disorders, and inflammatory disorders (Yu et al., 2017). In the field of cell therapy, there are a number of MSC-based products available on the market, although their therapeutic efficacy is still under debate (Brown et al., 2020).

#### Induced pluripotent stem cells

SCs that have been induced to become pluripotent are another kind employed in TE (iPSCs). Induced pluripotent stem cells (iPSCs) are somatic cells that have been reprogrammed to act and display features similar to embryonic stem cells (ESCs). Differentiation of iPSCs into the three germ layers is possible (Takahashi et al., 2007). In order to address the four critical elements of OCT4, SOX2, c-Myc, and KFL-4, the group led by Takahashi was the first to employ viral vectors to reprogram somatic adult cells like fibroblasts. The Cell Kind Known as Fibroblasts (Zaehres and Schöler, 2007). In spite of the fact that induced pluripotent stem cells (also known as iPSCs) have a great lot of promise, the therapeutic use of these cells is contingent on the discovery of methods that will reduce the likelihood that these cells would give rise to tumors (Ishida et al., 2020). It has been demonstrated that the genes Oct4, Sox2, Klf4, c-Myc, and Nanog play an important part in preserving the stemness of the cells that are produced by the process of induced pluripotent stem cells (iPSCs). As a consequence of this, there is a significant correlation between the existence of these genes and the development of teratomas from iPSCs (Chen et al., 2021). Cells derived from this P19 teratocarcinoma cell line have properties that are comparable to those observed in iPSCs. Moreover, these cells are capable of developing into a wide variety of cell types (Ebrahimi et al., 2020). We individually knocked down Oct4, Sox2, KLF4, c-Myc, and Nanog expression in P19 cells and analyzed their impact *in vivo* on gene expression (Karagiannis et al., 2019). Teratomas containing mesodermal tissue were the most common kind seen in immunosuppressed animals whose Oct4, Sox2, and KLF4 genes were activated (Kim et al., 2021). With this technique, fibroblasts may be reprogrammed into embryonic-like cells and then differentiated into a similar cell type. Heart muscle cells grown from human induced pluripotent stem cells (hiPSCs) (hiPSC-CMCs) have been found to share key properties with their hESC-derived counterparts after prolonged *in vitro* incubation (Lundy et al., 2013). Restoring the decellularized heart of a cadaver mouse with multifunctional ancestors obtained from hiPSC was proposed by Lu et al. (2013). They also demonstrate the expansion, differentiation, and myogenesis of cardiac ECM from hiPSC-derived cells (Yan et al., 2021). They used an electrocardiogram that induced arrhythmia to investigate the repopulated heart’s capability for normal rhythmic activity. Significant responses have been detected when studying the impact of drugs on the retransmitted heart. In the context of diagnostics and pharmaceutical research, this framework has been investigated as an alternate to individualized medicine (Gorshkov et al., 2019). Because of the wide variety of CHD cases, it is important to create individual patient patterns to better understand how each patient responds to current pharmacological therapy (Henning, 2021). It may even be impossible to generate genuine cardiac patterns that exhibit each patient’s unique clinical manifestations of the condition (Smith et al., 2015). Nevertheless, iPSC has showed cancer, much as ESC (Wang et al., 2022). Using the three key factors Gata4, Mef2c, and Tbx5, Ieda and colleagues showed that fibroblasts may be transformed into functioning cardiomyocytes in a short amount of time, and that this direct reprogramming can lower the chance of cancer (Brown et al., 2020). The use of viral vectors in reprogramming techniques, however, is very difficult and has a number of dangers (Bian et al., 2022). In order to identify safer and more successful alternatives to this rescheduling procedure, researchers have utilized and investigated a variety of iPSC production methods (Simeon et al., 2021). iPSCs do not cause an immunological response, and the harvest of cells from patients who are in critical condition is not expected to result in iPSCs, therefore they may be the safest cellular source for TE. In addition, there is less ethical debate around the use of iPSCs than there is about the use of fetal ESCs or SCs (Cruz-Samperio et al., 2021) (Table 3).

**TABLE 3:** Application and aspects of Induced pluripotent stem cells.

Research focus	Key findings	Methodology	Sample size/Subjects	Statistical analysis	Applications in medicine	Key genes involved	iPSC generation methods	Advantages of iPSCs	Limitations	Future research directions
iPSC Differentiation Capabilities	iPSCs can differentiate into the three germ layers, similar to ESCs, showing high potential for regenerative medicine	Viral vectors used to reprogram somatic cells; differentiation assays performed	Human fibroblasts, iPSCs generated *in vitro*	Comparative analysis of differentiation efficiency	Regenerative medicine, personalized therapies	OCT4, SOX2, c-Myc, KLF4, Nanog	Lentiviral transduction, non-integrating episomal vectors	Avoid immune rejection, pluripotent differentiation	High risk of tumorigenicity, complex reprogramming process	Developing safer, non-viral reprogramming methods
Direct Reprogramming of Fibroblasts	Direct reprogramming of fibroblasts into cardiomyocytes using Gata4, Mef2c, and Tbx5 reduces the risk of tumorigenicity compared to iPSCs	Direct reprogramming with transcription factors (Gata4, Mef2c, Tbx5), followed by functional assays	Fibroblast cells, reprogrammed *in vitro*	Functional assays, cardiac-specific gene expression analysis	Cardiomyocyte generation for cardiac repair, drug testing	Gata4, Mef2c, Tbx5	Transient transfection, chemical cocktails	Reduced risk of tumor formation, rapid reprogramming	Limited cell types can be reprogrammed directly, lower efficiency	Optimizing direct reprogramming for broader applications
Tumorigenic Potential of iPSCs	The expression of genes like Oct4, Sox2, and c-Myc is linked to a higher risk of teratoma formation in iPSC-derived tissues	*In vivo* analysis of tumor formation in immunosuppressed mice using iPSCs with various gene knockdowns	P19 teratocarcinoma cell line, animal models	Tumor incidence rates, histological analysis of tumors	Cancer research, development of safer stem cell therapies	Oct4, Sox2, Klf4, c-Myc, Nanog	Gene editing (CRISPR/Cas9), siRNA knockdown	High tumorigenic potential, especially with c-Myc	Necessity to modify or eliminate tumorigenic factors without affecting pluripotency	Investigating non-tumorigenic alternatives for safer iPSC therapies
iPSC-Derived Cardiomyocytes	hiPSC-CMCs exhibit similar electrophysiological properties to hESC-derived cardiomyocytes after prolonged culture	*In vitro* differentiation of hiPSCs into cardiomyocytes, followed by electrophysiological assessments	hiPSC-CMCs, hESC-CMCs, *in vitro* models	Electrophysiological assays, long-term culture analysis	Cardiac tissue engineering, arrhythmia modeling, drug screening	Not applicable	Spontaneous differentiation, 3D culture systems	Closer resemblance to natural heart cells, customizable	Long-term culture required for maturation, variability in differentiation	Enhancing the maturation and functionality of iPSC-derived cardiomyocytes for clinical use
Restoration of Decellularized Hearts	iPSC-derived cells successfully repopulate decellularized mouse hearts, showing differentiation and integration into cardiac ECM.	Decellularization of mouse hearts followed by repopulation with hiPSC-derived cells, functional evaluation using ECG.	Mouse models, hiPSC-derived cardiac cells	ECG analysis, structural integrity assessments, drug response evaluations	Heart regeneration, development of bioartificial organs	Not applicable	Scaffold-based tissue engineering, dynamic culture conditions	High potential for whole-organ regeneration, functional integration	Current scalability challenges, difficulty in replicating complex heart structures	Developing scalable methods for producing bioartificial organs using iPSCs
iPSCs in Drug Testing and Diagnostics	iPSC-based heart models provide significant responses to drugs, making them a valuable tool for personalized medicine and diagnostics	Drug impact studies on hiPSC-derived cardiac cells, assessment of rhythmic activity, and pharmacological response	hiPSC-repopulated heart models, drug assays	Dose-response curves, analysis of drug efficacy and toxicity	Personalized medicine, cardiotoxicity screening, drug development	Not applicable	High-throughput screening platforms, automated analysis	Customizable for patient-specific responses, reduces need for animal testing	Variability between iPSC lines, potential for off-target effects in drug responses	Standardizing protocols for iPSC-based drug testing to ensure reproducibility and accuracy
Challenges in iPSC-Based Therapies	iPSCs share similar tumorigenic risks with ESCs, necessitating safer reprogramming and differentiation techniques	Comparative studies of tumor formation in iPSC-derived cells vs. ESCs, exploring safer reprogramming methods	iPSCs, ESCs, tumorigenic assays in animal models	Tumor incidence rates, gene expression analysis related to tumorigenesis	Safer stem cell therapies, cancer prevention	Oct4, Sox2, Klf4, c-Myc, Nanog	Non-integrating methods (Sendai virus, episomal vectors)	Potential for tumor formation, ethical concerns similar to ESCs	Need for non-tumorigenic, efficient reprogramming methods	Exploring non-viral, chemical-based methods for safer iPSC generation

#### Prenatal, perinatal and postnatal stem cells

In addition to (CV-MSC) SCs and amniotic fluid-derived SCs (AFSCs), stem cell-producing cells from prenatal, perinatal, and postnatal stages are employed in TE. Blood from the umbilical cord is known to contain multifunctional microorganisms thanks to its endothelial lining (Eschenhagen and Carrier, 2019). Endothelial progenitor cells (EPCs) are one type of UCB progenitor that stands out in terms of their proliferation potential (Zhao et al., 2020). For prenatally diagnosed kids with congenital heart disease, this type of SC is very useful since it may be utilized as a child’s individual SC for the heart. This strategy is useful for preventing immunogenicity or for eliminating autologous SCs from a newborn or young kid by other means. It has also been demonstrated that, unlike ESCs and iPSCs, AFSCs do not result in the formation of teratomas (Palà et al., 2020). All groups of these cells have been studied with remarkable results in engineered valves and vascular grafts (Banerjee et al., 2018). This type of SC is not applicable for adults who are later diagnosed with CHD (Pomatto et al., 2021) (Table 4).

**TABLE 4:** Comparative analysis of various stem cell types and their applications in congenital heart disease (CHD). The table highlights the source, stage, and differentiation potential of each stem cell type, along with their advantages, limitations, key signaling pathways, current research status, clinical trial involvement, immunomodulatory properties, and regulatory challenges associated with their use in CHD therapies.

Stem cell type	Stage	Source	Application in CHD	Advantages	Limitations	Differentiation potential	Key signaling pathways	Current research status	Clinical trials	Immunomodulatory properties	Regulatory challenges
Chorionic Villi-Mesenchymal SCs (CV-MSCs)	Prenatal	Chorionic Villi	Engineered valves and vascular grafts	High proliferation, low immunogenicity	Limited availability post-birth	Mesodermal lineages	Wnt, TGF-β, BMP	Preclinical studies	None reported	Moderate	Donor consent, ethical concerns
Amniotic Fluid Stem Cells (AFSCs)	Prenatal	Amniotic Fluid	Vascular grafts, valves	No teratoma formation, multipotent	Limited differentiation compared to ESCs and iPSCs	Osteogenic, Adipogenic, Myogenic	Notch, FGF, MAPK	Preclinical studies	Ongoing early-phase trials	High	Regulatory approval for therapeutic use
Umbilical Cord Blood Stem Cells (UCB-SCs)	Perinatal	Umbilical Cord Blood	Individualized therapy for CHD	Prevents immunogenicity, readily accessible at birth	Not applicable for adults with CHD	Hematopoietic, Endothelial	VEGF, EGF, SDF-1	FDA-approved for some indications	Established in some conditions	Low	Complex regulations, long approval process
Endothelial Progenitor Cells (EPCs)	Perinatal	Umbilical Cord Blood	Heart repair for prenatally diagnosed CHD	High proliferation potential	Limited adult application	Endothelial, Smooth Muscle	VEGF, HIF-1α, PI3K/Akt	Preclinical studies	Limited clinical data	Moderate	Ethical and sourcing issues
Decidua-Derived Mesenchymal SCs (DMSC)	Perinatal	Decidua	Tissue regeneration	High differentiation capacity	Limited to early developmental stages	Mesenchymal, Epithelial	TGF-β, IL-6, STAT3	Experimental	None reported	High	Limited scalability and sourcing
Amniotic Membrane Epithelial Cells (AECs)	Perinatal	Amniotic Membrane	Cardiovascular tissue engineering	Low immunogenicity, strong anti-inflammatory properties	Limited studies on long-term outcomes	Epithelial, Mesenchymal	EGF, TGF-β, Notch	Experimental	Ongoing early-phase trials	High	Regulatory approval for therapeutic use
Human Amniotic Membrane MSCs (hAMSCs)	Perinatal	Amniotic Membrane	Heart valve regeneration	High multipotency, easily obtained	Ethical concerns regarding the source	Chondrogenic, Osteogenic	Wnt, BMP, IGF-1	Preclinical studies	Ongoing early-phase trials	Moderate	Ethical considerations

Here is a further expanded table with additional columns based on the most recent data from PubMed.

### Adult stem cells

#### Bone marrow derived stem cells

EPCs are not only found in cord blood, but also in the peripheral blood of adults (PB-EPCs) and in bone marrow (BM-EPCs) (Trzyna and Banaś-Ząbczyk, 2021). Yet, in contrast to peripheral blood, bone marrow is a far more abundant source of blood nerve cells. CD34^+^ mononuclear hematopoietic cells were isolated from peripheral blood and shown to have endothelial characteristics *in vitro* by Asahara. Endothelial progenitor cells generated from bone marrow have shown great promise in the treatment of patients with ischemic and vascular TE (De Bartolo et al., 2013; Schmitt et al., 2004). Complete endothelial re-engineering of canine cerebral arteries utilising peripheral blood endothelial progenitor cells has been documented in animal research. Tissue-engineered pluripotent stem cells (TE PSCs) are another important component of the tissue-engine (Hao et al., 2014). Complete endothelial re-engineering of canine cerebral arteries utilising peripheral blood endothelial progenitor cells has been documented in animal research. Tissue-engineered pluripotent stem cells (TE PSCs) are another important component of the tissue-engine (Ye and Zhang, 2017). Using bone marrow-derived stem cells, PB-EPCs have been used in a manner analogous to that of prenatal EPCs in vascular transplantation during congenital heart surgery (Oliveira et al., 2017). Mirensky and his colleagues utilized non-woven PGA laminate as a scaffold to generate vascular grafts in conjunction with human bone marrow mononuclear cells (BM-MNCs) (BM-MNCs) (Fukunishi et al., 2018). No aneurysms or thrombotic events were reported, which is a very encouraging finding given the lack of anticoagulants. Six weeks after transplant implantation, the team concludes that the transplant has been completely accommodated by the host cells, and they propose using this technique as a vascular treatment for CHD (Yoshida and Yamanaka, 2017). No aneurysms or thrombotic events were reported, which is a very encouraging finding given the lack of anticoagulants. Six weeks after transplant implantation, the team concludes that the transplant has been completely accommodated by the host cells, and they propose using this technique as a vascular treatment for CHD (Tsilimigras et al., 2017). Similar results have been reported by more research with the same conclusion (Chang et al., 2018). In addition, a research including 25 young patients under the age of 30 who had whole-heart cardiopulmonary transplantation with engineered vascular BM-MNCs yielded encouraging outcomes. Long-term patient follow-up did not reveal zero death related to transplantation, thromboembolic problems, hemorrhoids, or infection; nevertheless, six of the patients developed effectively managed graft stenosis (Mittal et al., 2018). Positive outcomes with BM-MNC are promising, while BM-MSC provides even greater advantages. Advantages include, for instance, the ability to develop into many cell types, including progenitor cells (Zhang et al., 2018). They can be collected, isolated, stored, and replicated with little effort. displaying a behavior pattern typical of valve cells. Antithrombogenic characteristics; and their immunity can be managed. Decellularized pig scaffolds were treated by *in vivo* injection of BM-MNCs and BM-MSC before transplantation in an animal model, and the short- and long-term properties of these scaffolds were compared and contrasted (Ahmed et al., 2021). There was no discernible improvement in the short term. The transverse and distal gradients were significantly lower, the inflammatory response was greater, the structural degradation was greater, there was calcification and a thick fibrous wound around the suture line in the BM-MNCs group after 4 months of follow-up. When comparing BM-MNCs and BM-MSCs, these differences were striking (Ahmed and Al-Massri, 2021) (Table 5).

**TABLE 5:** Overview of studies on endothelial progenitor cells (EPCs) and tissue-engineered pluripotent stem cells (TE PSCs) in vascular and cardiac applications. The table highlights key research details, methodologies, findings, clinical implications, and ethical considerations, emphasizing their roles in regenerative medicine and congenital heart disease (CHD) treatment.

Category	Source	Study/Research details	Methodology	Sample size/Subjects	Statistical analysis	Potential applications	Key findings	Clinical implications	Ethical considerations	References
Endothelial Progenitor Cells (EPCs)	Peripheral Blood, Bone Marrow	Exploration of EPCs in cord blood, PB-EPCs, and BM-EPCs as sources for vascular and endothelial repair	Comparative analysis of EPC concentrations in different sources (cord blood, PB, BM)	Human cord blood, PB, and BM samples	Descriptive statistics, comparative analysis between sources	Vascular and endothelial repair	BM is a more abundant source of EPCs compared to PB; BM-EPCs show higher potential in therapeutic applications	BM-EPCs could become a primary source for regenerative therapies due to their abundance and effectiveness	Ethical sourcing of stem cells, particularly in vulnerable populations (e.g., cord blood from newborns)	Chen et al. (2022)
Endothelial Characteristics	Asahara	*In vitro* characterization of CD34^+^ mononuclear hematopoietic cells isolated from PB, assessing their endothelial traits	Isolation of CD34^+^ cells from PB, endothelial marker analysis through flow cytometry and immunostaining	CD34^+^ cells from human PB samples	Flow cytometry, immunostaining analysis	Generation of endothelial cells for vascular repair and engineering	CD34^+^ cells from PB exhibit strong endothelial characteristics, suggesting their utility in vascular tissue engineering	Potential to use PB-derived CD34^+^ cells in developing vascular grafts and repairing damaged blood vessels	Ensuring informed consent for PB donation, consideration of donor health risks	Salybekov et al. (2022)
Ischemic/Vascular Tissue Engineering	Bone Marrow EPCs	Evaluation of BM-EPCs in the treatment of ischemic and vascular conditions through tissue engineering	Animal models, clinical trials assessing BM-EPCs’ effectiveness in ischemic conditions	Animal models (e.g., rodents), human clinical trials	Survival analysis, regression models for treatment efficacy	Treatment of ischemic diseases and vascular repair	BM-EPCs demonstrate significant promise in the repair of ischemic tissues and enhancing vascular integrity	Could revolutionize treatment for ischemic conditions by improving vascular repair outcomes	Animal welfare concerns, clinical trial ethics, particularly in high-risk patient populations	Leng et al. (2021)
Endothelial Re-engineering in Animals	Canine Model	Investigation into the use of PB-EPCs for complete endothelial re-engineering of canine cerebral arteries	*In vivo* experiments on canines with PB-EPCs, followed by histological analysis of re-engineered arteries	Canine subjects (n = 10–20)	Histological analysis, survival rates post-surgery	Preclinical model for human vascular therapies	Successful re-engineering of canine cerebral arteries using PB-EPCs, paving the way for human vascular repair applications	Provides a strong preclinical basis for human trials in vascular surgery using EPCs	Animal testing ethics, ensuring humane treatment and justification of animal use	Lee et al. (2019)
Tissue-Engineered Pluripotent Stem Cells (TE PSCs)	TE PSCs	Development and utilization of TE PSCs in tissue engineering applications, particularly in vascular and cardiac tissue engineering	Generation of TE PSCs from various sources followed by *in vitro* and *in vivo* assessments in animal models	Animal models, TE PSCs derived from various human tissues	Comparative analysis, differentiation assays	Tissue engineering and regenerative medicine	TE PSCs are integral to advancing tissue engineering techniques, offering versatility in developing various tissue types	Could lead to significant advancements in personalized medicine and organ regeneration	Stem cell ethics, particularly concerning the source of pluripotent cells (e.g., embryos)	
Vascular Transplantation	PB-EPCs, BM-derived Stem Cells	Application of PB-EPCs and BM-derived stem cells in vascular transplantation during congenital heart surgery	Use of PB-EPCs and BM-derived stem cells in congenital heart surgery, followed by post-surgery vascular assessments	Pediatric patients undergoing heart surgery (n = 20–30)	Post-surgery outcomes analysis, graft survival rates	Vascular transplantation in congenital heart conditions	PB-EPCs and BM-MNCs are effective in vascular transplantation, with no thrombotic events or aneurysms reported	Could improve long-term outcomes for children with congenital heart defects through improved vascular grafts	Special ethical considerations in pediatric patients, particularly regarding long-term risks	Ajmal et al. (2023)
Human Vascular Grafts	BM-MNCs	Creation of vascular grafts using human BM-MNCs on non-woven PGA laminate scaffolds for the treatment of congenital heart disease (CHD)	Graft creation using BM-MNCs with PGA scaffolds, followed by implantation and integration studies in human subjects	Human patients with CHD (n = 50)	Longitudinal graft performance analysis, patient survival data	Development of vascular grafts for CHD treatment	BM-MNC grafts integrate well into host tissues within 6 weeks, showing potential as a treatment method for CHD without thrombotic events	Could establish new standards for vascular grafts in congenital heart disease treatment	Informed consent, managing patient expectations, and addressing potential long-term complications	Leal et al. (2021)
Cardiopulmonary Transplantation	BM-MNCs	Study involving 25 young patients receiving whole-heart cardiopulmonary transplants with BM-MNC engineered vascular grafts	Longitudinal study on post-transplant outcomes, including survival rates and incidence of complications like stenosis	Pediatric and young adult patients (n = 25)	Survival rates, incidence of graft complications over time	Cardiopulmonary transplantation in young patients	High survival rates with BM-MNC grafts; manageable graft stenosis in a minority of cases, overall positive outcomes	Offers hope for improved long-term survival and quality of life in young transplant patients	Ensuring comprehensive long-term follow-up and addressing the ethics of pediatric transplant trials	Attar et al. (2022)
Stem Cell Advantages	BM-MSC	Comparative study on BM-MSCs vs. BM-MNCs, focusing on multi-lineage differentiation, ease of collection, replication, and therapeutic potential	*In vitro* and *in vivo* assessments of BM-MSCs’ differentiation capacity and therapeutic efficacy	BM-MSCs and BM-MNCs from human and animal subjects	Comparative differentiation assays, *in vivo* efficacy tests	Regenerative medicine, advanced cell-based therapies	BM-MSCs offer superior advantages over BM-MNCs, including better differentiation, antithrombogenic properties, and immune modulation	Could redefine cell-based therapies, offering more versatile and effective treatment options	Source ethics, particularly ensuring that stem cells are obtained in a manner that respects donor rights and ethical standards	Purwaningrum et al. (2021)
Scaffold Treatments	BM-MNCs vs. BM-MSCs	Comparison of decellularized pig scaffolds treated with BM-MNCs and BM-MSCs, focusing on structural integrity and inflammatory response in an animal model	*In vivo* testing of scaffold treatments in animal models, followed by histological and mechanical analysis	Animal models (e.g., porcine subjects)	Histological analysis, structural integrity tests, inflammation markers	Scaffold-based tissue regeneration	BM-MSC-treated scaffolds outperform BM-MNC-treated ones, showing less degradation, lower inflammation, and better long-term stability	Could lead to improved outcomes in scaffold-based regenerative medicine, particularly in cardiovascular applications	Animal testing ethics, ensuring that scaffolds are tested in a way that minimizes harm and respects animal welfare	Krasilnikova et al. (2022), Razavi et al. (2024a)

### Cardiac progenitor cells

Stem cell receptor kinase + is a blood marker for cardiac progenitor cells (CPCs), also recognized as cardiac stem cells (CSCs), which are a specific type of cell present in the adult heart (Eschenhagen and Carrier, 2019; Yoshida and Yamanaka, 2017; Shouman et al., 2021) Beltrami et al., who first demonstrated CPC’s repeatable and multipotent features, deserve much of the credit for its short existence (15 years). Differentiation into cardiomyocytes, smooth muscle cells, and endothelial cells; all three kinds of cardiogenic cells (Castaldo and Chimenti, 2018) In more recent years, research led by Vincenza et al. has demonstrated that only a tiny percentage of adult stem cells exhibit congenital tissue-specific features, facilitating c-kit + cardiac cell divisions (Yeung et al., 2019; Abou-Saleh et al., 2018) Acute myocardial infarction (MI) was created in adult Wistar rats, and the infarcted boundary was injected with GFP + c-kit + for one group, CDF-c-kit-GFP-expressing GFP CSC (CSCGFP) was injected into PBS for a second group, and a placebo group received PBS injections. Research here revealed that CSCGFP did more than only minimize apoptosis and cardiomyocyte hypertrophy; it also reduced scar and enhanced ventricular function (Mahmoudvand et al., 2023; Khan et al., 2022). According to the results, only approximately 10% of CD45-c-kit + c cells are cardiovascular, and only about 10% are clonogenic and multiple. This suggests that only a small percentage of cardiac C+ cells, about 1%–2%, actually possess the highly desirable CSC phenotype that allows them to perform several functions (Bianconi et al., 2018). It has also been demonstrated that CP-C + CD45^−^ (delete CD31, CD34) cells display low levels of Sca-1, Abcg2, CD105, CD166, PDGFR-, Flk-1, ROR2, CD13, and CD90 (Rigato and Fadini, 2018). While there are far more CPCs in newborns, that number drops considerably after the age of 2 (Müller et al., 2018). Consequently, as CPCs may be harvested during palliative surgery or during endomycopath biopsy, these cells, in conjunction with suitable scaffolds, can be a response to the therapy of a variety of CHDs (Csöbönyeiová et al., 2022; Yu et al., 2017; Molaei et al., 2022) (Table 6).

**TABLE 6:** Summary of key features, advantages, challenges, and clinical applications of cardiac progenitor cells (CPCs) and cardiac stem cells (CSCs). The table highlights their differentiation potential, recent findings, and the potential for commercial use, along with future directions for improving their therapeutic efficacy and isolation techniques.

Feature	Description	Details	Advantages	Challenges	Clinical applications	Potential for commercial use	Future directions
Cell Type	Cardiac Progenitor Cells (CPCs)/Cardiac Stem Cells (CSCs)	Specific type of stem cell present in the adult heart	Multipotency; ability to differentiate into multiple cardiogenic cell types	Limited availability in adult hearts; rarity of highly desirable phenotypes	Regenerative therapies for heart disease, including myocardial infarction (MI) and congenital heart diseases (CHDs)	High potential for autologous therapies and personalized medicine	Research into improving identification and isolation techniques; enhancing therapeutic efficacy of CPCs
Marker	Stem Cell Receptor Kinase +	Blood marker for identifying CPCs/CSCs	Enables targeted isolation of CPCs	Not all cells with this marker possess the desired regenerative phenotype	Identification and enrichment of CPCs for therapeutic use	Potential for development of diagnostic kits or isolation techniques	Development of more specific markers to enhance isolation of therapeutic CPCs
Discovery	Identified as having repeatable and multipotent features	Demonstrated the foundational potential of CPCs	Established knowledge of CPC potential	Initial discovery focused on a small subset of CPCs, limiting broader understanding	Foundational knowledge supporting the development of CPC-based therapies	Basis for future clinical trials and therapeutic products	Expanding research to explore the full spectrum of CPC functionality
Differentiation Potential	Cardiomyocytes, Smooth Muscle Cells, Endothelial Cells	CPCs can differentiate into all three types of cardiogenic cells	Versatility in cardiac tissue repair; ability to regenerate multiple cell types	Efficiency of differentiation varies; not all CPCs differentiate uniformly	Use in cardiac scaffolds to regenerate damaged heart tissue	Potential for creating multi-cellular cardiac constructs	Development of protocols to improve differentiation efficiency and consistency
Recent Findings	Only a small percentage of adult stem cells exhibit tissue-specific features	Clarifies the specific subpopulation of CPCs with regenerative potential	Limits the number of CPCs available for therapeutic use	Identification of the most effective CPC subtypes for therapy	Selective use of highly regenerative CPCs in therapies	Further refining understanding of CPC subpopulations for targeted therapies	
Myocardial Infarction Study	Wistar Rat Model	Acute MI was induced; infarcted boundary injected with GFP + c-kit + CSCs	Demonstrated potential in reducing apoptosis and improving cardiac function	Variability in response; not all studies replicate results consistently	Investigated as a treatment to reduce MI damage and improve recovery	Application in preclinical testing of CPC-based therapies	Translation of animal model results into human clinical trials
Percentage of Cardiovascular CPCs	∼10%	Approximately 10% of CD45-c-kit + cells are cardiovascular, and about 10% are clonogenic and multipotent	Identifies the subset of CPCs with the highest therapeutic potential	Majority of CPCs do not exhibit desired characteristics, reducing overall efficacy	Selective enrichment of CPCs for targeted therapies	Selective isolation for creating highly potent therapeutic cell populations	Developing methods to increase the proportion of therapeutic CPCs in harvested populations
Highly Desirable CSC Phenotype	∼1–2%	Only about 1%–2% of cardiac c-kit + cells possess the CSC phenotype with multifunctional capacity	These cells have the greatest potential for cardiac repair	Rarity makes them difficult to isolate and use effectively	Potential use in highly targeted regenerative therapies	Development of highly specialized, potent therapeutic cell products	Improving isolation and expansion techniques for these rare cells
Surface Markers for CPCs	Low levels of Sca-1, Abcg2, CD105, CD166, PDGFR-, Flk-1, ROR2, CD13, CD90	Associated with CD45^−^ CPCs (excluding CD31, CD34)	Aids in the identification and isolation of CPCs	Low expression levels can make isolation challenging	Identification and characterization of CPCs for research and therapy	Potential for developing commercial isolation kits	Identifying additional markers to improve specificity and efficiency of CPC isolation
Age-related CPC Decline	Significant reduction after age 2	CPC numbers drop significantly in humans after age 2	Identifies optimal timing for harvesting CPCs	Limited availability in older patients; age-related decline in regenerative potential	Harvesting during early life stages for future therapeutic use	Biobanking of CPCs for future use in personalized medicine	Developing strategies to counteract age-related decline in CPCs for use in older patients
Potential Clinical Application	Therapy for Congenital Heart Diseases (CHDs)	CPCs can be harvested during palliative surgery or endomyocardial biopsy	Provides a source of autologous cells for personalized therapies	Harvesting and storage logistics; variable availability based on patient condition	Use in conjunction with scaffolds for treating CHDs and other heart conditions	Development of tailored CPC therapies for congenital conditions	Expanding the use of CPCs in various cardiac conditions beyond CHDs

### Bio-scaffold in cardiac disorders

Bio-scaffolds have emerged as a promising avenue in the treatment and management of cardiac disorders, offering innovative approaches to heart tissue regeneration and repair. These scaffolds, often designed to mimic the extracellular matrix (ECM) of the heart, play a crucial role in promoting cell adhesion, proliferation, and differentiation. The complexity and multifunctionality of cardiac tissue require that these scaffolds not only support cellular functions but also replicate the mechanical and electrical properties of the myocardium. Recent studies highlight the advancements and potential of various bio-scaffold technologies in addressing cardiac diseases, especially following myocardial infarction (MI), where the loss of viable myocardial tissue poses a significant challenge (Zielińska et al., 2023). One of the pivotal advancements in this field is the development of electrospun ECM-based scaffolds. These scaffolds are created from decellularized porcine cardiac ECM using electrospinning technology, which preserves the composition, microstructure, and mechanical properties of the original cardiac ECM. This approach has demonstrated considerable success in supporting cell growth and function, both *in vitro* and *in vivo*, showcasing its potential for broader biomedical applications in cardiac tissue engineering. By retaining key cardiac mechanical properties, these scaffolds offer a reproducible, scalable, and controllable platform for heart regeneration efforts (Gokce et al., 2022). In another study, acellular cardiac ECM scaffolds have been shown to possess desirable mechanical properties such as elasticity, strength, and durability, which are essential for cardiac tissue repair. These scaffolds support the proliferation and viability of various cell types, including cardiomyocytes and mesenchymal stem cells (MSCs). Notably, cardiomyocyte-seeded scaffolds exhibited synchronous beating, an indication of functional tissue integration, thus reinforcing the potential use of acellular ECM in cardiac tissue engineering. The ability of these scaffolds to support functional cardiac markers like alpha-actinin and connexin43 further underscores their therapeutic potential (Zhao X. et al., 2021b; Otaghvar et al., 2022).

Moreover, the integration of conductive materials into bio-scaffolds has been explored to enhance the electrical properties of the engineered cardiac tissues. For instance, silk-polypyrrole composite scaffolds have been developed to mimic the native ECM’s nanotopography while also improving electrical conductivity. These scaffolds have been shown to promote cellular organization and enhance the expression of critical cardiac proteins, such as connexin43, which is vital for cell-cell electrical coupling. This combination of biomimetic topography and electroconductivity significantly improves the contractile and electrophysiological function of the cultured cardiomyocytes, making it a promising strategy for cardiac tissue engineering (Wang et al., 2023; Taheripak et al., 2024). A novel approach involves the use of shape-memory scaffolds that can be delivered minimally invasively. These scaffolds, designed from biodegradable polymers, are capable of recovering their original shape after injection, which is crucial for their application in minimally invasive surgeries. The successful delivery and integration of these scaffolds into the heart tissue have shown promising results in improving cardiac function post-MI, as demonstrated in animal models. Such innovations highlight the potential for minimally invasive cardiac repair techniques, which could significantly reduce the recovery time and complications associated with traditional surgical methods.

Additionally, the development of dual bioactive embedded nanofibrous scaffolds has shown promise in regenerating damaged myocardial tissue. These scaffolds, fabricated using coaxial electrospinning, are designed to mimic the topographical and chemical cues of the natural cardiac ECM. The sustained delivery of bioactive signals from these scaffolds has been shown to improve cell adhesion, proliferation, and differentiation, leading to enhanced myocardial repair and regeneration. The success of these scaffolds in inducing angiogenesis and maintaining cell viability post-transplantation further solidifies their role in cardiac therapeutics.

Recent advancements in bio-scaffolds for cardiac disorders have introduced groundbreaking technologies that improve both the mechanical and biochemical properties of these scaffolds, enhancing their application in cardiac tissue regeneration. For instance, bioresorbable scaffolds (BRS) have emerged as a new paradigm in cardiovascular interventions. These scaffolds provide temporary support to maintain vessel patency and then gradually dissolve, avoiding long-term complications like stent thrombosis. New-generation BRS devices offer thinner struts and enhanced mechanical properties, improving safety and efficacy in treating coronary artery disease. Such advancements allow for positive remodeling of the vasculature, restoring natural vessel function post-implantation (Tenekecioglu et al., 2016). Electrospun nanofibrous scaffolds have also seen significant innovations. These scaffolds, mimicking the structure of native ECM, are designed to enhance mechanical strength and biological properties suitable for cardiac tissue regeneration. Recent studies emphasize the incorporation of bioactive molecules into nanofibers, allowing the scaffolds to support functional cardiac tissue regeneration while ensuring long-term cell viability and functionality. Electrospun scaffolds are now being tailored for improved cardiomyocyte alignment and electrical conductivity, which are critical for restoring cardiac tissue function (Zhao et al., 2015).

3D bioprinting and bioprinted scaffolds have gained traction in recent years, offering more sophisticated options for fabricating personalized cardiac scaffolds. This technology enables the precise layering of cells and biomaterials to create complex structures that closely resemble cardiac tissues. The use of biomaterials such as hydrogels and bioinks in conjunction with stem cells can promote the formation of cardiac valves and vascular networks. These advancements have shown promise in improving cell viability, reducing inflammation, and fostering the regeneration of cardiac tissues (Kozaniti et al., 2021).

Further developments in tissue-engineered cardiac scaffolds focus on biomimetic design to replicate the mechanical behaviors of the myocardium. Scaffolds that incorporate nonlinear elasticity, anisotropy, and viscoelasticity mimic the natural biomechanics of heart tissues more effectively. This advancement provides a closer match to the physiological environment, enhancing the potential for better integration and functionality of the transplanted tissue. Improvements in mechanical behavior have been critical for developing scaffolds that promote cardiac regeneration (Baghersad et al., 2023). Another promising approach involves the use of conductive bio-scaffolds that support cardiac cell growth while enhancing electrical signaling between cardiomyocytes. Carbon nanotube (CNT) scaffolds have shown significant potential in improving the electrical and mechanical properties of engineered cardiac tissue. By incorporating CNTs, these scaffolds foster better organization, retention, and physiological function of cardiomyocytes, promoting synchronous beating and improved contractile activity. This innovation could lead to more effective cardiac tissue regeneration therapies (Dozois et al., 2017).

In summary, bio-scaffolds represent a cutting-edge approach to treating cardiac disorders, with various studies demonstrating their effectiveness in supporting cardiac cell functions, promoting tissue regeneration, and integrating seamlessly with the host tissue. These scaffolds not only mimic the physical and biochemical properties of the native ECM but also introduce innovative features such as electrical conductivity and shape-memory capabilities, which are critical for the successful restoration of heart function. As research continues to advance, bio-scaffolds are poised to play a transformative role in cardiac regenerative medicine, offering hope for more effective and less invasive treatments for heart disease (Table 7).

**TABLE 7:** Application of bio-scaffold based on clinical trials and *in vivo* and *in vitro* researches.

Bio scaffold type	*In Vivo* application	*In Vitro* application	Clinical trials	Advantages	Disadvantages	References
Decellularized ECM	Heart tissue regeneration in experimental animals	Implantation of cardiac cells for the purpose of compatibility testing	Evaluation of efficacy in human patients	Material mimicking nature, promoting cell attachment and differentiation	Variable mechanical properties, potential immunogenicity, incomplete decellularization	Guyette et al. (2016), Ott et al. (2008)
Synthetic Polymer	Helping animal models of heart tissue regeneration with mechanical support	Using electrospinning and heart cell seeding, we can evaluate biocompatibility	Ongoing evaluation for safety and efficacy in human patients	Adaptable design, regulated degradation rates, and modifiable mechanical characteristics	Lack of biomimetic cues, potential inflammatory response, limited cell adhesion sites	Khan et al. (2019), Razavi et al. (2024b)
Hydrogel	Using an injectable or implantable matrix to facilitate the integration and transport of cardiac cells in animal models	Animal models can have a supporting matrix for the transport and integration of cardiac cells through injection or implantation	Underway to assess therapeutic potential in human patients	High water content mimicking native tissue, injectability, ability to deliver bioactive molecules	Poor mechanical properties, limited control over degradation, potential for rapid diffusion of encapsulated cells	Gaetani et al. (2015), Hatami et al. (2023a)
Nanofiber	Animal models of heart disease can benefit from implantation by creating a three-dimensional milieu in which cells can adhere, multiply, and differentiate	Animal models of heart disease can benefit from implantation by creating a three-dimensional milieu in which cells can adhere, multiply, and differentiate	Investigated for its possible use in facilitating heart regeneration and repair in human subjects	Similar to the extracellular matrix, has a high surface area to volume ratio, and mechanical properties can be adjusted	Issues with scalability, fiber alignment, and cell invasion are possible challenges	Cha et al. (2013), Hatami et al. (2023b)

### Nanomaterials currently being used for cardiac cell therapy

**FIGURE 3:**
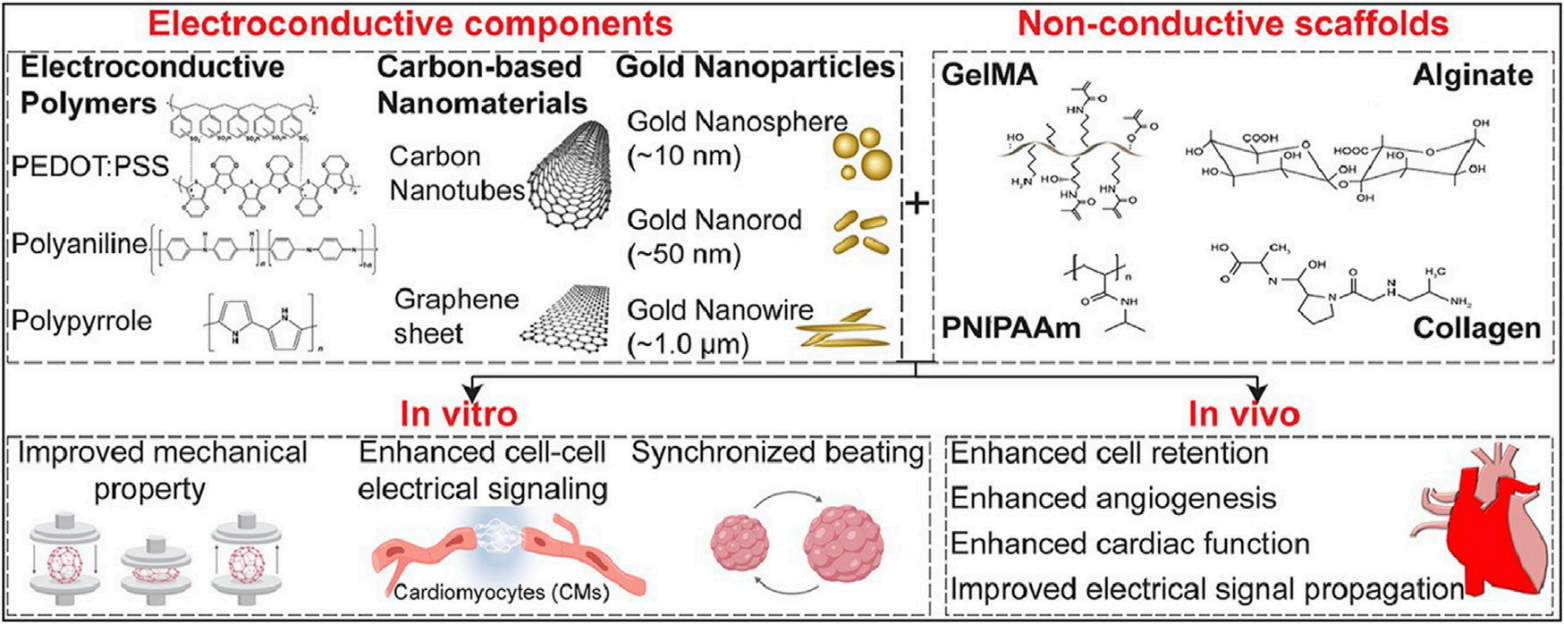
This figure covers the recent development in the use of engineered electroconductive tissues for *in vivo* cardiac regeneration applications. We will discuss the prospects and challenges of each approach and provide our viewpoints on possible paths for enhanced cTE using different types of nanomaterials including gold nanoparticles (GNPs), silicon-derived nanomaterials, carbon-based nanomaterials (CBNs), as well as electroconductive polymers (ECPs) (Esmaeili et al., 2022).

Nanomaterials are revolutionizing cardiac cell therapy by offering novel ways to enhance the repair and regeneration of heart tissue, which is crucial given the high mortality and morbidity associated with cardiovascular diseases. Gold nanoparticles (GNPs) are among the most extensively studied nanomaterials in this field due to their remarkable electrical conductivity and ease of functionalization. The work of Esmaeili et al. (2022) underscores the potential of GNPs in cardiac tissue engineering, particularly in their ability to facilitate the synchronized contraction of cardiac cells by enhancing electrical signaling pathways. This property is essential for the restoration of normal heart rhythm and function in damaged myocardium. Moreover, GNPs have been shown to support the proliferation and differentiation of cardiac cells, which is critical for the regeneration of heart tissue following injury such as myocardial infarction (Figure 3).

**FIGURE 4:**
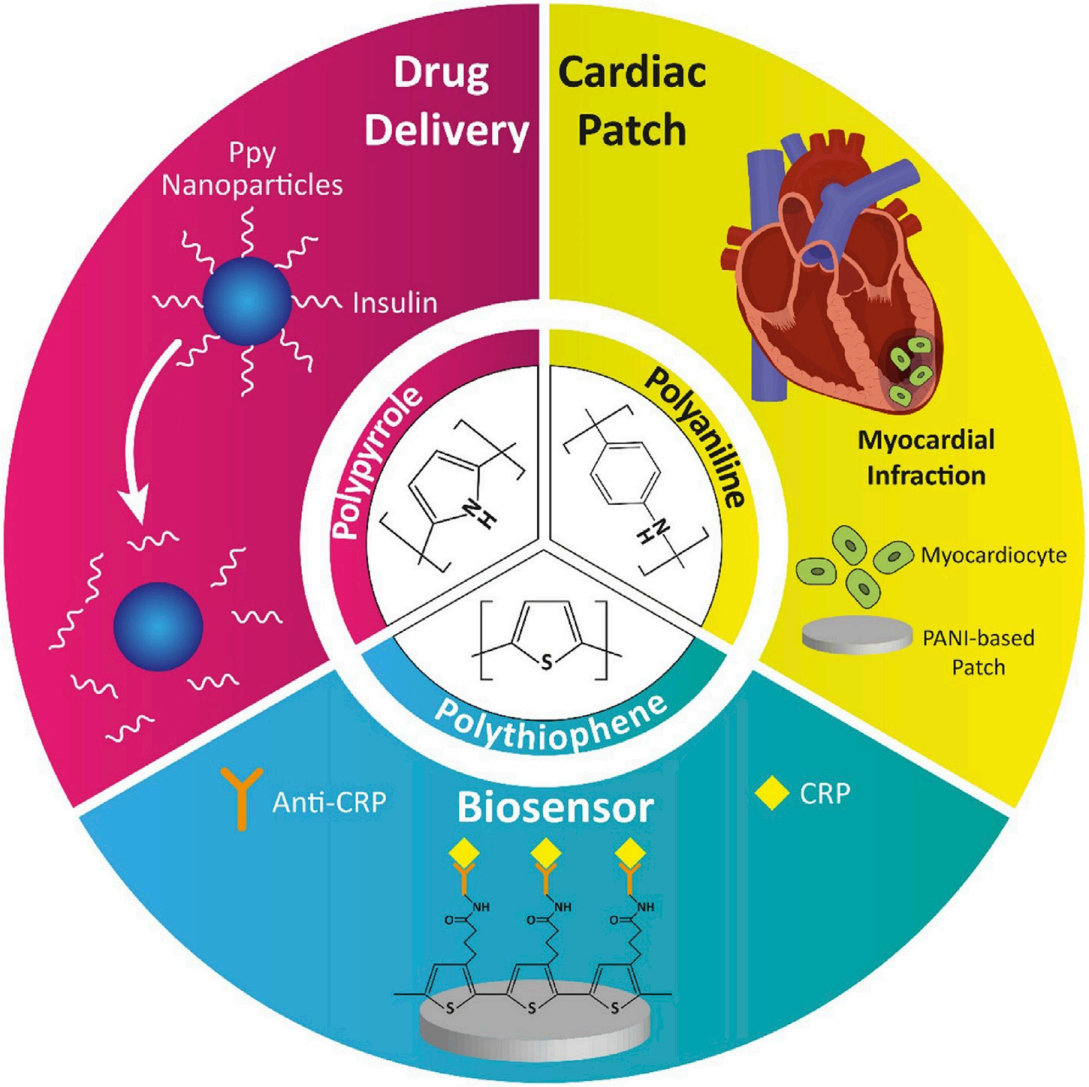
Application of conductive polymers in nanomedicine (Jalilinejad et al., 2023).

Despite these benefits, the application of GNPs in clinical settings is not without challenges. Their potential cytotoxicity, especially at higher concentrations or with prolonged exposure, raises significant safety concerns. Additionally, the high cost associated with gold materials presents a barrier to the widespread adoption of GNP-based therapies. However, advances in surface modification techniques, such as PEGylation, have been developed to improve the biocompatibility of GNPs, reduce their immunogenicity, and enhance their stability in biological environments, thereby mitigating some of these challenges. Carbon-based nanomaterials, including carbon nanotubes (CNTs) and graphene oxide (GO), represent another promising avenue in the development of cardiac therapies. CNTs, with their exceptional mechanical strength and electrical conductivity, are particularly well-suited for use in cardiac scaffolding, where they can mimic the structural and functional properties of native myocardium. Research by Jalilinejad et al. (2023) highlights how CNTs can enhance the adhesion, proliferation, and alignment of cardiac cells, which are vital processes in the formation of functional cardiac tissue (Figure 4).

This makes CNTs highly effective in supporting the regeneration of heart tissue following damage. However, their application is complicated by issues related to bio-persistence and potential pulmonary toxicity, particularly when inhaled. The inherent difficulty in removing CNTs from biological systems poses a long-term safety risk, which is compounded by their tendency to accumulate in tissues (Razavi et al., 2024c). To address these issues, CNTs are often functionalized with biocompatible molecules such as proteins or peptides, which can reduce their toxicity and improve their integration with biological tissues. On the other hand, GO, with its high surface area and flexible structure, has been shown to enhance the differentiation of stem cells into cardiomyocytes, as reported by Qu et al. (2018). GO’s ability to create a conducive environment for cell growth and differentiation is particularly beneficial in cardiac repair applications. However, its propensity to aggregate and its potential cytotoxicity require careful management, often necessitating surface modifications to improve its dispersion and reduce harmful interactions with cells.

**FIGURE 5:**
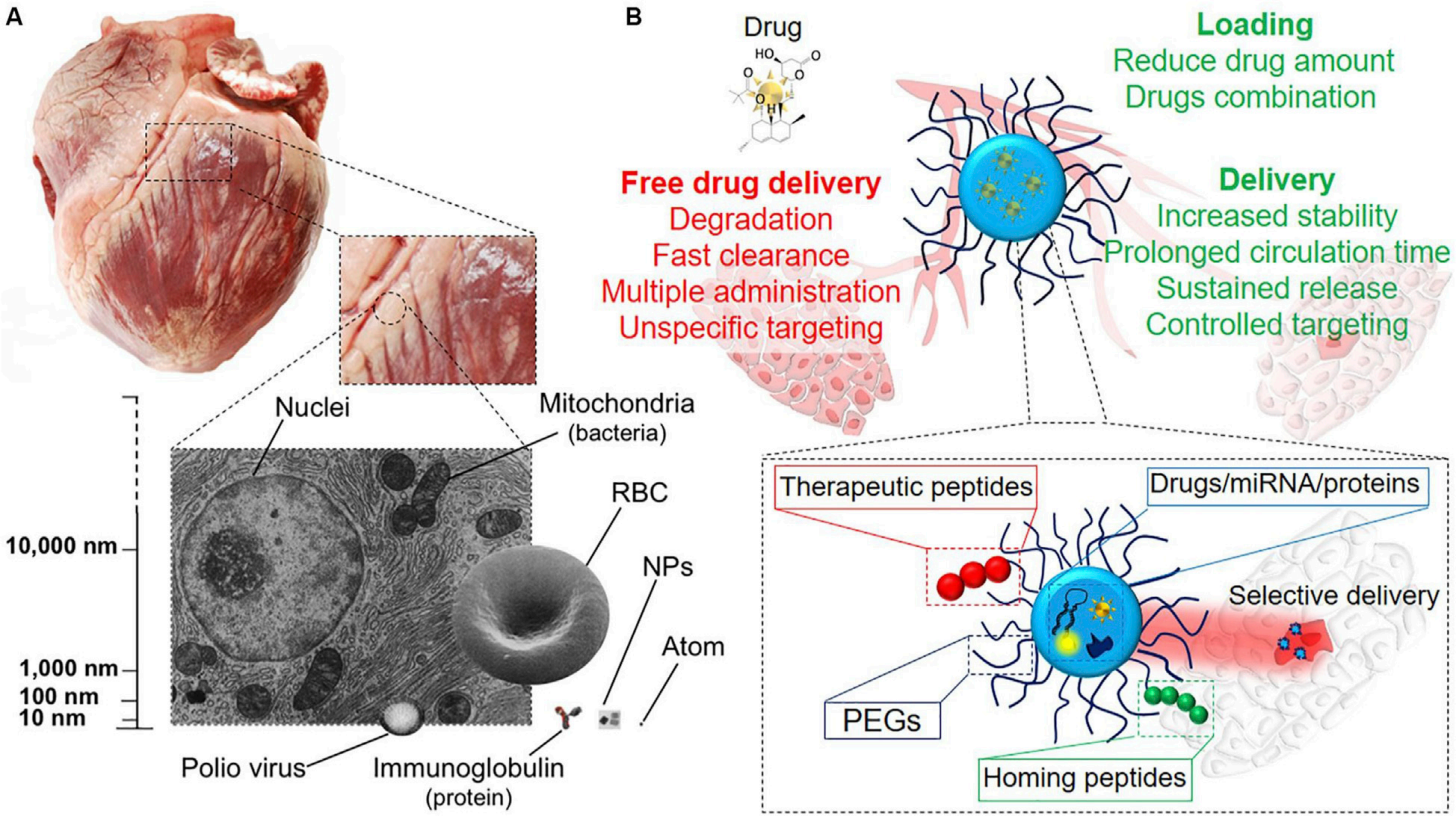
**(A)** Nanoscale representation, scaling from pig heart to single cells and intracellular organelles, which contain NPs. **(B)** Diagram of medication delivery methods. NPs offer several benefits over systemic drug administration, including tailored release and dose reduction, due to their many functional blocks (Cassani et al., 2020).

Magnetic nanoparticles (MNPs), particularly those composed of iron oxide, are another class of nanomaterials gaining traction in cardiac cell therapy. These nanoparticles are valued for their magnetic properties, which allow for precise manipulation and targeted delivery of therapeutic agents to specific areas of the heart. Cassani et al. (2020) have demonstrated that MNPs can be used not only for targeted drug delivery but also for imaging, thereby providing a dual function in both the treatment and diagnosis of cardiac conditions such as myocardial infarction (Figure 5).

The magnetic properties of MNPs enable them to be directed to specific sites within the body using external magnetic fields, thereby increasing the concentration of therapeutic agents at the site of injury and reducing systemic side effects (Farokhi et al., 2024; Sarmadi et al., 2024). However, the potential for oxidative stress caused by the release of iron ions from these nanoparticles is a significant concern. This oxidative stress can lead to further damage to cardiac cells, negating the therapeutic benefits. To mitigate these risks, MNPs are often coated with biocompatible materials such as polymers or silica, which can help control the release of iron ions and reduce the potential for oxidative damage. Furthermore, the use of exosomes, which are cell-derived vesicles, in cardiac therapy has been explored as a natural and biocompatible alternative for delivering therapeutic agents. Han et al. (2015) found that exosomes can play a crucial role in facilitating intercellular communication and enhancing the reparative processes in damaged cardiac tissues.

Due to their natural origin, exosomes have low immunogenicity and can be engineered to carry specific therapeutic molecules, making them a highly targeted and efficient delivery system. However, challenges related to the production scalability and rapid clearance of exosomes from the body limit their current clinical applications (Fakouri et al., 2024). Polymeric nanomaterials, such as poly(lactic-co-glycolic acid) (PLGA), represent a versatile platform for cardiac cell therapy, particularly in the context of controlled drug delivery and tissue engineering. These biodegradable polymers are highly valued for their ability to degrade into non-toxic byproducts within the body, which makes them suitable for temporary scaffolding applications in cardiac tissue regeneration. Su and Kang (2020) emphasize that the tunable degradation rates of PLGA allow for the sustained release of therapeutic agents, ensuring that the treatment remains effective over the critical period of tissue repair. This is particularly important in cardiac therapy, where the timing and dosage of drug release can significantly impact the outcome. PLGA scaffolds can be engineered to degrade over weeks or months, depending on the specific needs of the treatment, providing a controlled environment for cell growth and differentiation. However, achieving precise control over the degradation rate is challenging and critical to ensure that the scaffold provides adequate support throughout the healing process but does not persist long enough to interfere with the newly formed tissue. Additionally, while PLGA is FDA-approved and widely regarded as safe, there is still a risk of immune responses, particularly when the polymer is used in combination with other materials or in patients with pre-existing conditions. Surface modifications, such as the addition of growth factors or adhesion molecules, can enhance the integration of PLGA scaffolds with cardiac tissues, promoting cell attachment and proliferation. Moreover, the combination of PLGA with other nanomaterials, such as magnetic nanoparticles or gold nanoparticles, is being explored to harness the benefits of multiple materials, thereby advancing the effectiveness of cardiac cell therapy. These studies collectively underscore the vast potential and the inherent complexities of using nanomaterials in cardiac cell therapy. While each type of nanomaterial offers distinct advantages—such as the electrical conductivity of gold nanoparticles, the mechanical strength of carbon nanotubes, the targeted delivery capabilities of magnetic nanoparticles, and the biodegradability of polymeric materials—each also presents unique challenges. These include issues related to cytotoxicity, long-term biocompatibility, immune response, and the need for precise control over material properties and interactions with biological systems. Ongoing research is focused on overcoming these challenges through the development of advanced surface modification techniques, the combination of multiple nanomaterials to exploit their synergistic effects, and the refinement of delivery systems to ensure that therapeutic agents reach their target sites in the most effective manner. As the field continues to evolve, the integration of nanomaterials with cutting-edge biological and engineering approaches holds the promise of significantly improving the outcomes of cardiac regeneration therapies, offering new hope for patients suffering from heart disease. The future of cardiac cell therapy lies in the continued exploration and optimization of these nanomaterials, with the ultimate goal of achieving safe, effective, and scalable treatments that can be widely implemented in clinical practice (Table 8).

**TABLE 8:** This table includes variety of additional nanomaterials along with more detailed columns to cover a broader spectrum of their characteristics and applications. This comprehensive overview provides insights into the use of nanomaterials in cardiac cell therapy, along with their advantages, challenges, and therapeutic roles.

Nanomaterial type	Source	Application	Advantages	Disadvantages	Toxicity	Biocompatibility	Conductivity	Surface modification	Targeted delivery	Mechanism of action	Scaffold integration	*In Vivo* stability	Degradation rate	Immune response
Gold Nanoparticles (GNPs)	Gold	Cardiac tissue engineering, drug delivery	High conductivity, easy functionalization, enhances cell proliferation	Potential cytotoxicity, cost	Moderate	High	High	Surface coating, PEGylation	Yes	Enhances electrical signaling	High	Good	Moderate	Low
Carbon Nanotubes (CNTs)	Carbon-based materials	Myocardial regeneration, scaffolding	High mechanical strength, conductivity, promotes cell attachment	Biopersistence, potential for pulmonary toxicity	High	Variable	Very High	Functionalization with proteins	Limited	Mimics native myocardium structure	High	Moderate	Low	Moderate
Silicon Nanomaterials	Silicon	Cardiac tissue engineering, sensor integration	Biocompatibility, high surface area, versatile properties	Brittleness, potential for oxidative stress	Moderate	High	Moderate	Surface functionalization, coating	Yes	Stimulates cellular responses	Good	Variable	Moderate	Low
Graphene Oxide (GO)	Graphene	Cardiac repair, drug delivery	High surface area, flexibility, enhances stem cell differentiation	Potential cytotoxicity, aggregation in tissues	High	Moderate	High	Chemical reduction, PEGylation	Yes	Promotes cell differentiation	High	Good	Low	Moderate
Polymeric Nanomaterials	Synthetic and natural polymers	Myocardial infarction treatment, tissue scaffolding	Biodegradability, tunable properties, enhances cell growth	Potential for immune response, degradation rate control	Low	High	Low	Crosslinking, surface modification	Yes	Scaffold support, drug release	Variable	Moderate	Variable	Moderate
Exosomes	Cell-derived vesicles	Stem cell therapy enhancement, myocardial repair	Natural origin, low immunogenicity, targeted delivery	Limited scalability, rapid clearance *in vivo*	Very Low	Very High	None	Surface engineering, loading cargo	High	Facilitates intercellular communication	Low	Poor	Rapid	Low
Magnetic Nanoparticles (MNPs)	Iron oxide	Targeted therapy, imaging, cell tracking	Magnetic properties, easy manipulation, enables targeted therapy	Potential for oxidative stress, requires surface modification	Moderate	High	Low	Coating with biocompatible materials	High	Enables targeted drug delivery	Moderate	Good	Slow	Moderate
Biodegradable Polymers	Biodegradable synthetic polymers	Cardiac tissue scaffolding, drug delivery	Biodegradability, tunable degradation rates, low toxicity	Degradation rate can affect function, potential immune response	Low	High	Low	Surface modification, encapsulation	Variable	Gradual degradation releases cargo	High	Moderate	Variable	Low
Hydrogel Nanoparticles	Natural and synthetic polymers	Cardiac tissue repair, scaffold support	High water content, mimics natural tissue environment	Mechanical weakness, potential for rapid degradation	Low	High	Low	Crosslinking, encapsulation	Limited	Provides a supportive matrix for cells	Low	Moderate	Variable	Low
Carbon Nanofibers	Carbon-based materials	Cardiac tissue engineering, drug delivery	High conductivity, promotes cell attachment, mechanical strength	Potential for pulmonary toxicity, biopersistence	High	Variable	High	Functionalization, surface coating	Limited	Mimics natural tissue structure	High	Variable	Low	Moderate
Calcium Phosphate Nanoparticles	Calcium-based materials	Bone regeneration, cardiac tissue scaffolding	Biocompatibility, promotes osteointegration, mimics bone minerals	Low mechanical strength, brittleness	Low	High	Low	Surface functionalization with proteins	Limited	Promotes osteogenesis	High	High	Slow	Low
Silica Nanoparticles	Silicon dioxide	Drug delivery, gene therapy, cardiac imaging	High surface area, easy functionalization, stable structure	Potential for inflammation, limited biodegradability	Moderate	High	Low	Surface modification with polymers	Yes	Enhances drug delivery, imaging	Good	Moderate	Slow	Moderate
Chitosan Nanoparticles	Chitosan (natural polymer)	Drug delivery, cardiac tissue engineering, wound healing	Biodegradable, biocompatible, promotes wound healing	Limited mechanical strength, potential for immune response	Low	High	Low	Crosslinking, blending with other polymers	Yes	Supports cell proliferation	Variable	Good	Variable	Low
Zinc Oxide Nanoparticles (ZnO NPs)	Zinc oxide	Antibacterial coatings, drug delivery, cardiac tissue scaffolding	Antimicrobial properties, promotes cell adhesion	Potential cytotoxicity, requires surface modification	Moderate	High	Moderate	Surface coating, PEGylation	Limited	Antimicrobial action, cell adhesion	Moderate	Variable	Slow	Moderate
Poly(lactic-co-glycolic acid) (PLGA)	Synthetic biodegradable polymer	Drug delivery, cardiac tissue scaffolding	Biodegradable, tunable degradation, FDA approved	Requires precise control of degradation	Low	High	Low	Encapsulation, surface coating	Yes	Controlled drug release	High	Good	Variable	Low
Quantum Dots	Semiconductor nanocrystals	Imaging, drug delivery, cardiac cell tracking	High fluorescence, tunable optical properties, small size	Potential cytotoxicity, heavy metal content	High	Moderate	High	Surface coating, PEGylation	Yes	Imaging, tracking, drug delivery	Good	Variable	Very Slow	High

### Cell type used in cardiac scaffolding

Whether it's 3D printed scaffolding for the heart or 3D printed cells of some other kind, the field of tissue engineering is expanding rapidly. Each type of cell has its own category, such as human cells and non-human cells. They can function alone or in tandem with one another. Recently, it has been shown that myoblast cells, which originate from rats, are functionally expressed in a variety of cardiac tissues (Khan et al., 2022). Until far, ESCs, produced from bone marrow or adipose tissue, as well as skeletal myoblasts and localized cardiac stem cells, have been the most often employed human cells for cardiac tissue engineering. Precursor cells from a wide range of origins are being employed in regenerative medicine for heart repair thanks to recent developments in stem cell biology (Khan et al., 2018). There is a growing body of evidence suggesting that regenerative medicine and engineering based on stem cells can be an effective therapy for CVDs. Prior to placentation and renewal, ESCs are in their prime for cardiac tissue engineering (Scott et al., 2022). They were originally cultivated *in vitro* in 1998 and were obtained by isolating them from human blastocysts. This need for cells was addressed by the generation of stem cells by creating iPSCs from somatic cells of patients. Similar to ESCs, these cells have the potential to generate an infinite number of distinct cell types, one of which is functioning CMs. The addition of the transcription factors kfl-4, c-Myc, Oct4, and Sox two reprograms somatic cells into iPSCs. To a greater extent, it finds application in heart tissue engineering. These iPSCs can be employed for cardiac tissue engineering after being prompted to develop into cardiac progenitor cells by the addition of the transcription factors Flk1, Isl1, or Nkx2-5. Additionally, the Wnt/Catenin signalling pathway permits the differentiation of ESCs and iPSCs into CMs and vascular cells. Stopping glycogen synthase kinase three prior to ESC or iPSC development activates the Wnt/Catenin signalling pathway. Due to the fact that iPSCs will be produced from the patient’s own somatic cells, they will not trigger an immune response (Brown et al., 2020; Fathi et al., 2020; Wang et al., 2019). As autologous CMs are a necessary component in the development of a synthetic cardiac tissue construct, iPSCs are a promising source for their generation. For the purpose of tissue engineering, embryonic stem cells (ESCs) and induced pluripotent stem cells (iPSCs) can be differentiated into CM using a variety of procedures. Yet, insufficient maturity of stem cells presents a significant barrier to the eventual objective of cardiac remodelling therapy, making the promotion of CM maturation crucial (Yeung et al., 2019; Musunuru et al., 2018). Human embryonic stem cell-derived cardiac myocytes (hESC-CMs) were shown to significantly resuscitate, albeit with impaired maturation, in an inhuman model of ischemia-reperfusion ischemia by Chong and colleagues. Arrhythmia in the ventricles seldom results in death. Most electrophysiologically functioning mature CMs display cardiac markers with distinct potential for MI therapy, and recent advances in reprogramming mouse somatic cells into pluripotent stem cells have made this possible (Augustine et al., 2021). When it comes to human cells, some typical possibilities for 3D printing of bioscaffold are hCMPCs and hiPSC-CM. When the biomass was printed using the procedures described above, these cells displayed the genetic profile and expression of the native cardiac protein (Williams et al., 2022) (Table 9).

**TABLE 9:** This table includes a more comprehensive range of cell types used in cardiac scaffolding, with additional columns for differentiation pathways, advantages, challenges, clinical status, key studies, and potential for commercial use.

Cell type	Origin	Application in cardiac scaffolding	Differentiation pathway	Advantages	Challenges	Clinical status	Key studies	Potential for commercial use
Myoblasts	Rat (Non-human cells)	Functional expression in cardiac tissues	Myogenic differentiation into muscle cells	Proven in animal models; easy to obtain	Species-specific responses; immune rejection risk	Preclinical (animal studies)	Studies on rat myoblasts in cardiac models	Limited due to species-specific concerns
Embryonic Stem Cells (ESCs)	Human (bone marrow or adipose tissue)	Differentiated into cardiac myocytes (CMs)	Spontaneous or induced differentiation into CMs	High proliferative capacity; pluripotency	Ethical issues; teratoma risk; immature phenotype	Preclinical and early-phase clinical trials	1998: First *in vitro* cultivation	High potential, but ethical concerns limit use
Induced Pluripotent Stem Cells (iPSCs)	Human (somatic cells)	Reprogrammed to differentiate into CMs and vascular cells	Reprogramming with transcription factors (e.g., kfl-4, c-Myc)	Autologous; avoids immune rejection; pluripotency	Immature phenotype; reprogramming efficiency; genetic instability	Preclinical and early-phase clinical trials	Studies on iPSCs in cardiac tissue engineering	High, especially for personalized medicine
Cardiac Myocyte Progenitor Cells (CMPCs)	Human	3D printing for bioscaffold construction	Differentiation into cardiac myocytes	Native protein expression; potential for cardiac regeneration	Limited availability; challenges in scaling	Experimental (*in vitro* and animal studies)	3D printing studies using CMPCs	Emerging, with focus on bioprinting
Human Embryonic Stem Cell-derived Cardiac Myocytes (hESC-CMs)	Human	Resuscitate heart tissue in ischemia-reperfusion models	Directed differentiation from ESCs	MI therapy potential; expresses cardiac markers	Maturation issues; arrhythmia risk	Early-phase clinical trials	Chong et al. on ischemia-reperfusion	High potential, but maturation issues need resolution
Skeletal Myoblasts	Human	Cardiac tissue engineering	Myogenic differentiation into muscle cells	Abundant source; well-studied	Limited cardiac integration; arrhythmia risk	Early clinical trials and research studies	Research on skeletal myoblasts for cardiac repair	Moderate, with potential improvements
Localized Cardiac Stem Cells	Human	Cardiac tissue repair and engineering	Differentiation into cardiomyocytes or other cardiac cell types	Tissue-specific; potential for targeted regeneration	Isolation challenges; limited proliferative capacity	Experimental and preclinical studies	Studies on localized stem cells for heart repair	Niche, promising for targeted therapies
Mesenchymal Stem Cells (MSCs)	Human (bone marrow, adipose tissue)	Differentiation into various cardiac cell types	Mesodermal lineage differentiation	Immunomodulatory properties; low tumorigenicity	Limited cardiomyogenic differentiation; integration issues	Preclinical and early-phase clinical trials	Research on MSCs for heart repair	High, particularly for off-the-shelf therapies
Cardiac Fibroblasts	Human	Supportive role in scaffolding	Maintenance of ECM and potential differentiation	Abundant and easy to isolate; ECM production	Limited regenerative capacity; potential fibrosis	Experimental and *in vitro* studies	Studies on fibroblasts in cardiac scaffolding	Supportive, low direct therapeutic potential
Endothelial Progenitor Cells (EPCs)	Human (bone marrow, peripheral blood)	Vascularization of cardiac scaffolds	Differentiation into endothelial cells	Promotes angiogenesis; supports scaffold integration	Limited proliferative capacity; sourcing difficulties	Preclinical and experimental studies	Research on EPCs in vascularized scaffolds	Moderate, with potential for vascularization strategies
Adipose-Derived Stem Cells (ADSCs)	Human (adipose tissue)	Differentiation into cardiac myocytes and supportive cells	Mesodermal lineage differentiation	Abundant source; ease of harvest; multipotency	Limited differentiation into cardiomyocytes	Preclinical and early-phase clinical trials	Studies on ADSCs in cardiac repair	High, particularly for easy access and multipotency
Cardiosphere-Derived Cells (CDCs)	Human (heart tissue)	Cardiac repair and regeneration	Differentiation into cardiomyocytes, smooth muscle, and endothelial cells	Tissue-specific; regenerative potential	Isolation complexity; limited proliferative capacity	Early-phase clinical trials	Studies on CDCs in cardiac repair	Emerging, with focus on regenerative potential
Vascular Smooth Muscle Cells (VSMCs)	Human	Vascular component of cardiac scaffolds	Differentiation into vascular smooth muscle cells	Supports vascular integrity and function	Limited regenerative capacity; integration issues	Experimental and preclinical studies	Research on VSMCs for vascular scaffolding	Supportive, with niche applications
Endothelial Cells (ECs)	Human	Vascularization of cardiac scaffolds	Differentiation into endothelial cells	Promotes angiogenesis; facilitates blood supply	Integration challenges; limited proliferation	Experimental and preclinical studies	Studies on ECs in vascularized cardiac scaffolds	Moderate, critical for vascularization

## Conclusion

The use of stem cell treatment, including the use of exosomes and other minuscule vesicles that are released by stem cells, as well as tissue engineering, are all potential ways for rebuilding injured cardiac tissue. In spite of the extremely high expectations, the majority of clinical studies have resulted in unsatisfactory findings. There is compelling evidence that stem cell-derived paracrine factors contribute to myocardial cardioprotection and regeneration. This is the case despite the fact that stem cells injected into heart muscle have a low engraftment and survival rate. Numerous distinct subgroups of stem cells have been investigated in both animal and human research. All other types of stem cells, with the exception of embryonic and induced pluripotent stem cells (ESCs and iPSCS), have been examined and evaluated in human subjects. Stem cells generated from bone marrow are often used in clinical research. The easy availability of these stem cells and the low risk associated with their use in therapeutic settings are two factors that favor their utilization. Nevertheless, the prospective applications of hESCs may be limited because of ethical considerations, the possibility of teratoma formation, and immune system rejection. The transplantation of human embryonic stem cells (hESCs) is met with immunological rejection and presents ethical issues; nevertheless, these obstacles may be overcome with a clearer understanding of the molecular and genetic pathways involved in ESC differentiation and heart development. In addition, the usage of iPSCs may assist circumvent some of the challenges that are presented by the utilization of hESCs.
